# Multi‐parametric MRI synthesis for glioblastoma from quantitative MR fingerprinting: Quantitative synthetic neural network (QS‐Net)

**DOI:** 10.1002/mp.70435

**Published:** 2026-04-12

**Authors:** Yimin Ni, Chenyang Liu, Wen Li, Ling Lin, Rushan Ouyang, Peng Cao, Ho‐Fun Victor Lee, Aya El Helali, Yat‐Lam Wong, Xiang Wang, Peilin Wang, Ge Ren, Jing Cai, Tian Li

**Affiliations:** ^1^ Department of Health Technology and Informatics The Hong Kong Polytechnic University Hung Hom Hong Kong SAR China; ^2^ Department of Radiology The Eighth Affiliated Hospital Sun Yat‐sen University Shenzhen China; ^3^ Department of Diagnostic Radiology The University of Hong Kong Pok Fu Lam Hong Kong SAR China; ^4^ Department of Clinical Oncology The University of Hong Kong Pok Fu Lam Hong Kong SAR China; ^5^ Department of Clinical Oncology Queen Mary Hospital Pok Fu Lam Hong Kong SAR China

**Keywords:** contrast synthesis, magnetic resonance fingerprinting, model generalizability

## Abstract

**Background:**

Diagnosis and treatment of glioblastoma (GBM) rely on multiparametric MRI (mpMRI), but mpMRI is time‐consuming and costly. Deep learning–based synthesis methods have been proposed to streamline acquisition; however, their generalizability is limited by variability in qualitative input contrasts across sites and scanners.

**Purpose:**

To overcome this limitation, we developed and evaluated a generalizable deep learning model that synthesizes mpMRI contrasts directly from quantitative magnetic resonance fingerprinting (MRF) maps in GBM patients. The proposed Quantitative Synthesis Network (QS‐Net) employs a deeply supervised residual U‐Net generator within an adversarial framework, combined with a two‐stage training strategy to separate anatomical and pathological learning.

**Methods:**

We collected MRF‐derived T1 and T2 maps, along with conventional mpMRI sequences (T1w, T2w, T1‐FLAIR, T2‐FLAIR, and SWI), from 32 healthy volunteers and retrospectively from 18 GBM patient scans. The proposed QS‐Net was initially trained on healthy volunteer data (20 scans for training, 12 for testing) to learn general anatomical features. Subsequently, it was fine‐tuned using 9 GBM patient scans to adapt to pathological characteristics, with the remaining 9 patient scans reserved for independent testing. We compared the performance of QS‐Net against three state of the art deep learning models: Res‐Unet, conditional GAN, and Swin‐Transformer, using both quantitative metrics (MAE, SSIM, and PSNR) and qualitative assessments. Additionally, we assessed the generalizability of the models by evaluating their external validation performance when trained with either conventional MRI or quantitative MRF inputs.

**Results:**

QS‐Net outperformed the comparison models in synthesizing T1w, T2w, SWI, and T2‐FLAIR images for GBM patients, achieving the best results across all quantitative metrics: MAE (1.18 ± 0.52, 1.01 ± 0.36, 1.05 ± 0.37, 1.45 ± 0.76), SSIM (0.934 ± 0.037, 0.939 ± 0.039, 0.934 ± 0.034, 0.926 ± 0.053), and PSNR (29.69 ± 3.21, 29.35 ± 2.29, 29.64 ± 2.58, 27.56 ± 3.39), respectively. Qualitative analysis demonstrated that QS‐Net generated synthetic images with superior resemblance to ground truth, accurately delineating tumor boundaries and preserving intra‐tumoral texture. Furthermore, the generalizability test revealed that models trained on standardized quantitative MRF input maps consistently outperformed models trained on vendor‐specific qualitative MRI inputs across all architectures and metrics (*p* < 0.005).

**Conclusions:**

We developed QS‐Net, a deep learning model for high fidelity mpMRI synthesis from quantitative MRF maps, and demonstrated that this quantitative‐input paradigm enables superior cross‐vendor generalization over conventional qualitative MRI‐based approaches.

## 1 INTRODUCTION

Glioblastoma (GBM) represents the most frequently occurring primary malignant brain tumor, making up 16% of all primary brain and central nervous system neoplasms.^1^ The age‐adjusted incidence rate of GBM is approximately 3.2 cases per 100,000 individuals.^2^ The median overall survival for patients diagnosed with GBM is approximately 12 to 18 months. Survival rates remain low, with only 25% of patients surviving beyond one year and less than 5% achieving survival beyond five years.^3^ The infiltrative and heterogeneous nature of GBM underscores the need for accurate detection and diagnosis for better patient care. In this scenario, multiparametric magnetic resonance imaging (mpMRI) serves as the cornerstone technology for GBM management, integrating T1‐weighted (T1w), T2‐weighted (T2w), fluid attenuation inversion recovery (FLAIR), and susceptibility‐weighted imaging (SWI) sequences. Each of these sequences provides distinct and complementary insights. Diagnostically, the T1w and T2w imaging delineates tumor core and necrosis, while T2‐FLAIR demonstrates > 85% sensitivity for peritumoral edema.^4^ SWI effectively detects microhemorrhages and abnormal vascular proliferation.^5^ Prognostically, MRI radiomic features (e.g., intratumoral heterogeneity, infiltrative margins) predict molecular subtypes and survival rates.^6^ For treatment monitoring, morphological MRI are also pivotal in pinpointing treatment‐related changes from tumor progression.^7^

While each sequence provides distinct and complementary insights, the necessity for multiple acquisitions extends scanning duration, thereby increasing costs and patient discomfort. As a clinical study pointed out, a standard brain MR protocol consists of 6–8 distinct sequences. On average, a single sequence scan takes 3–5 min, and the total scan time adds up to 20–30 min per patient.^8^ The prolonged image acquisition increases the chance of patient discomfort, which leads to patient movement and motion artifacts of the acquired image. As summarized by a retrospective review, motion artifacts in MRI scanning led to repeated sequences in 19.8% of examinations, resulting in potential institutional costs of approximately $115,000 per scanner annually due to forgone revenue.^9^

In response to these limitations, deep learning (DL)‐based multiparametric MRI synthesis has been proposed to generate multiple contrast images from a single scan to minimize redundant acquisitions. Leveraging convolutional neural networks (CNNs), generative adversarial network (GAN), or self‐attention mechanism‐based Transformer architectures, this technology integrates baseline sequences (e.g., T1 and T2) to decode tissue relaxation properties and pathological signatures, synthesizing advanced contrasts such as T2‐FLAIR, proton density map (PD), and apparent diffusion coefficient (ADC). The state of the art synthetic models proposed between 2019 and 2023 have shown promising results: a structural similarity index (SSIM) of 0.9 or greater was achieved for the generation of multiple missing sequences, including T1w, T2w, T2‐FLAIR, and PD.^10^, ^11^, ^12^, ^13^, ^14^, ^15^, ^16^, ^17^ These technical advancements highlight the potential to enhance clinical efficiency and patient comfort using DL methods. However, existing models rely on qualitative MRI inputs, which can lead to inconsistencies in prediction accuracy across different facilities. In addition, no generalizability comparison has been conducted using multi‐institutional data.

Magnetic resonance fingerprinting (MRF) is an innovative quantitative imaging technique that acquires core tissue property maps (e.g., T1 and T2) within a single, rapid, and standardized acquisition. Unlike conventional qualitative MRI, MRF's dictionary‐matching reconstruction provides intrinsic normalization against a physics‐based model, enhancing reproducibility across scanners and sites.^18^, ^19^, ^20^ This combination of efficiency and inherent standardization makes MRF particularly compelling as a source of input data for building generalizable deep learning models. In prostate cancer, for instance, MRF‐derived T1/T2 maps complement ADC values, providing comprehensive tissue characterization with reduced acquisition time compared to conventional quantitative mapping approaches.^21^ MRF has demonstrated utility across various diseases, correlating with histologic grading in liver disease^22^ and quantifying tissue properties for breast cancer,^23^ cartilage assessment,^24^ brain tumor differentiation,^25^ and cardiac tissue mapping.^26^ While broad commercial deployment across all MRI vendors is ongoing, these studies establish MRF's core technical strength: its ability to provide reproducible quantitative maps that are less sensitive to scanner‐specific variations than conventional qualitative MRI. This makes it a powerful candidate source of data for generalizable deep learning.

The qualitative nature of conventional MRI introduces interscanner and interprotocol variability. Such variability critically undermines the generalizability of deep learning models across heterogeneous imaging environments. To overcome these obstacles, we propose a novel, generalizable mpMRI synthesis framework leveraging single‐sequence quantitative MRF. We validated our model's superiority over conventional MRI‐based approaches using multi‐institutional datasets, demonstrating enhanced anatomical consistency and diagnostic utility in heterogeneous imaging environments.

## 2 METHODS

### 2.1 Study design

**FIGURE 1:**
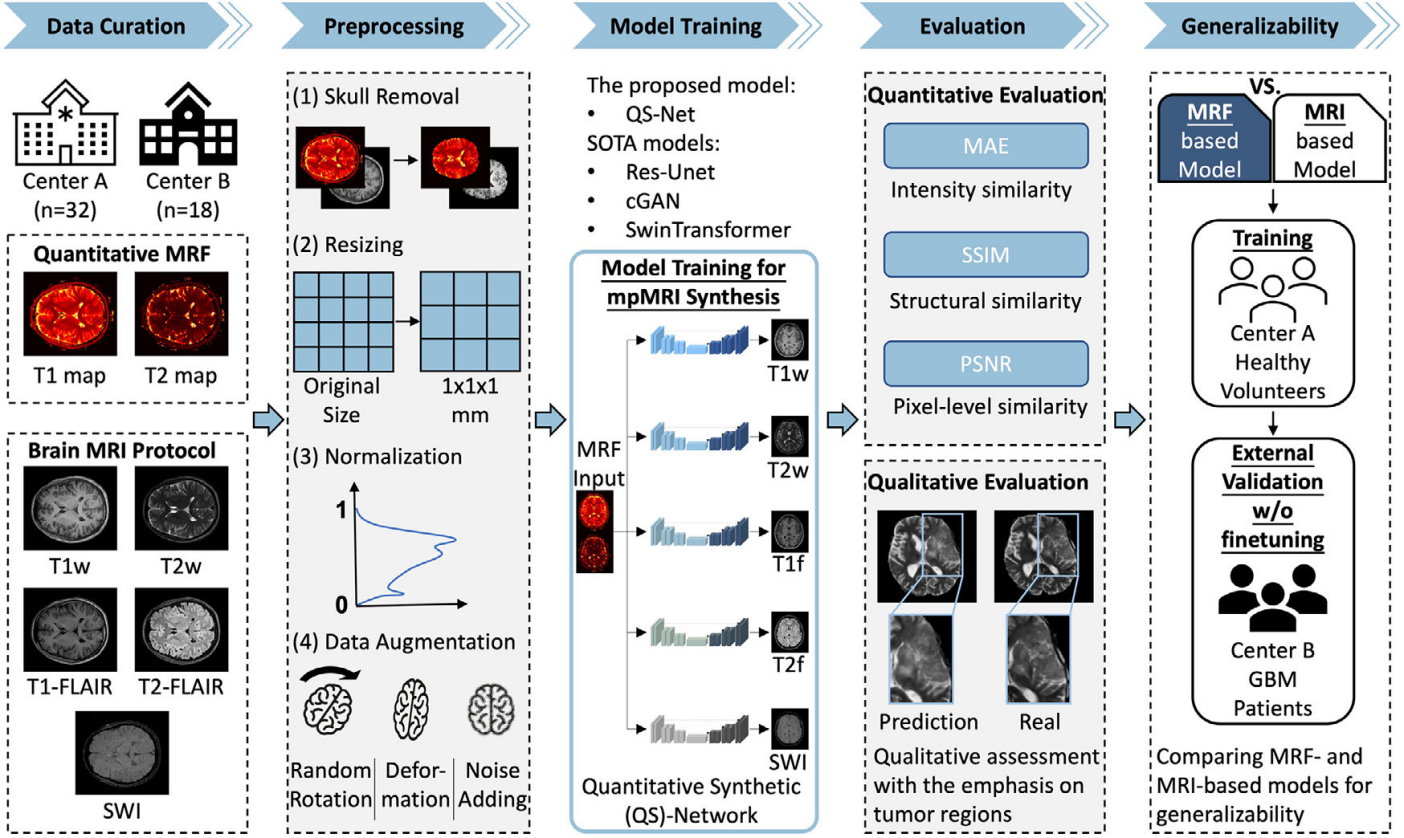
Schematic overview of the deep learning workflow. (a) **Data curation**: MRF T1 and T2 maps and multiparametric MRI were collected from 32 healthy volunteers and 18 GBM patients across two centers. (b) **Data preprocessing**: Skull removal, image resizing, normalization, and augmentation were performed to ensure data quality and consistency for supervised learning. (c) **Model training**: The proposed quantitative synthetic network (QS‐Net) and three state of the art models—Res‐UNet, conditional GAN (cGAN), and Swin Transformer—were trained and compared. (d) **Model evaluation**: Performance was assessed using mean absolute error (MAE), structural similarity index (SSIM), and peak signal‐to‐noise ratio (PSNR), along with qualitative visual inspection. (e) **Generalizability testing**: Models trained with either conventional MRI or quantitative MRF inputs were evaluated for external validation performance to assess generalizability.

Figure 1 illustrates the overall workflow of the study, which comprises five main components: data curation, data preprocessing, model training, model evaluation, and a comparison of generalizability between MRF‐based and MRI‐based models. Detailed descriptions of each component are provided below.

### 2.2 Patient data

The MRF T1 and T2 maps, together with multiparametric MRI, including T1w, T2w, T1‐FLAIR, T2‐FLAIR, and SWI, were collected from 32 healthy volunteers and were retrospectively retrieved from 18 GBM patient scans who received treatment at xxx Hospital between 2022 and 2024. This study was ethically and scientifically reviewed and approved by the Institutional Review Board (IRB) committee, and written informed consent was obtained from all healthy volunteers prior to their inclusion in the study. The requirement for informed consent from patients was waived due to the retrospective nature of the study. The MRF and mpMRI of healthy volunteers were acquired with a 3T MAGNETOM Prisma, Siemens Healthineers (Erlangen, Germany) scanner (referred to as Center A). The images of GBM patients were acquired with a 3T SIGNA Premier, GE Healthcare (Chicago, IL, USA) scanner (referred to as Center B). Detailed acquisition parameters for all MRF and conventional mpMRI sequences—including specific pulse sequence names and key parameters—are provided in Table 1.

**TABLE 1:** Acquisition parameters for magnetic resonance fingerprinting (MRF) and multi‐parametric MRI (mpMRI) sequences.

	Center A (3T MAGNETOM Prisma, Siemens Healthineers (Erlangen, Germany))					
	MRF	T1w	T2w	T1‐FLAIR	T2‐FLAIR	SWI
Sequence name	MRF	MPRAGE	TSE	T1‐TSE‐IR	T2‐SPACE	SWI
TR (ms)	12	2300	5500	2000	7000	27
TE (ms)	∖	2.32	117	8.3	395	20
TI (ms)	21	900	∖	900	2050	
Flip angle (FA)	Variable	8°	90°	150°	Variable	15°
Echo train length (ETL)	∖	∖	14	45	865	
Slice thickness (mm)	5	0.9	4	4	0.9	1.5
Pixel spacing (mm)	1.0 × 1.0	0.94 × 0.94	0.8 × 0.8	0.8 × 0.8	0.9 × 0.9	0.86 × 0.86
FOV (mm)	256 × 256	240 × 240	256 × 256	256 × 256	256 × 256	256 × 256


	Center B (3T SIGNA Premier, GE Healthcare (Chicago, IL, USA))					
	MRF	T1w	T2w	T1‐FLAIR	T2‐FLAIR	SWI
Sequence name	MRF‐FISP	BRAVO	PROPELLER	FSE‐IR	3D CUBE FLAIR	SWAN
TR (ms)	Variable	8.7	6347	2554	6300	39.6
TE (ms)	∖	4.3	120	25.6	105	24.2
TI (ms)	18	450	∖	802	1809	
Flip angle (FA)	Variable	12°	160°	150°	Variable	15°
Echo train length (ETL)	∖	∖	30	8	180	
Slice thickness (mm)	5	0.5	5.5	0.6	0.6	1.5
Pixel spacing (mm)	1.17 × 1.17	0.47 × 0.47	0.45 × 0.45	0.45 × 0.45	0.47 × 0.47	0.45 × 0.45
FOV (mm)	256 × 256	240 × 240	230 × 230	230 × 230	240 × 240	230 × 230

Abbreviations: ETL, Echo Train Length; FA, Flip Angle; FOV, Field of View.;MRF, Magnetic Resonance Fingerprinting; SWI, Susceptibility‐Weighted Imaging; T1‐FLAIR, T1‐weighted Fluid‐Attenuated Inversion Recovery; T1w, T1‐weighted; T2‐FLAIR, T2‐weighted Fluid‐Attenuated Inversion Recovery; T2w, T2‐weighted; TE, Echo Time; TI, Inversion Time; TR, Repetition Time.

### 2.3 Image pre‐processing

In preparation for deep learning training, all MRI images underwent a comprehensive preprocessing pipeline to ensure data quality and consistency. Firstly, the mpMRIs were coregistered to the corresponding MRF quantitative maps using rigid registration with the Elastix toolbox.^27^ Following this, skull stripping was performed to extract only the brain tissue, eliminating nonrelevant anatomical structures and reducing potential noise in the data. Next, each image was resized to a standardized dimension, facilitating a uniform input size for the neural network. Intensity normalization was then applied to adjust the pixel values to a range of [0,1]. Finally, data augmentation was employed to increase the diversity of the training data and mitigate overfitting. Specifically, random rotations (up to ± 15°) were applied to simulate variation in head orientation.^28^ Random elastic deformations were applied using a dense displacement field with a standard deviation of 2 pixels to introduce plausible anatomical variability, a technique widely used in biomedical image analysis.^29^ Gaussian random noise (zero mean, standard deviation equal to 1% of the image intensity range) was also added to simulate acquisition artifacts and improve model robustness.^30^ These transformations were applied on the fly during training.

### 2.4 Model architecture and implementation details

The Quantitative Synthesis Network (QS‐Net) proposed in this study is a GAN specifically designed for high fidelity, generalizable synthesis of mpMRI from quantitative MRF maps. Its novelty lies in two principal components: (1) a hybrid generator architecture and (2) a two‐stage, pathology‐aware training strategy.

#### 2.4.1 Hybrid generator architecture

**FIGURE 2:**
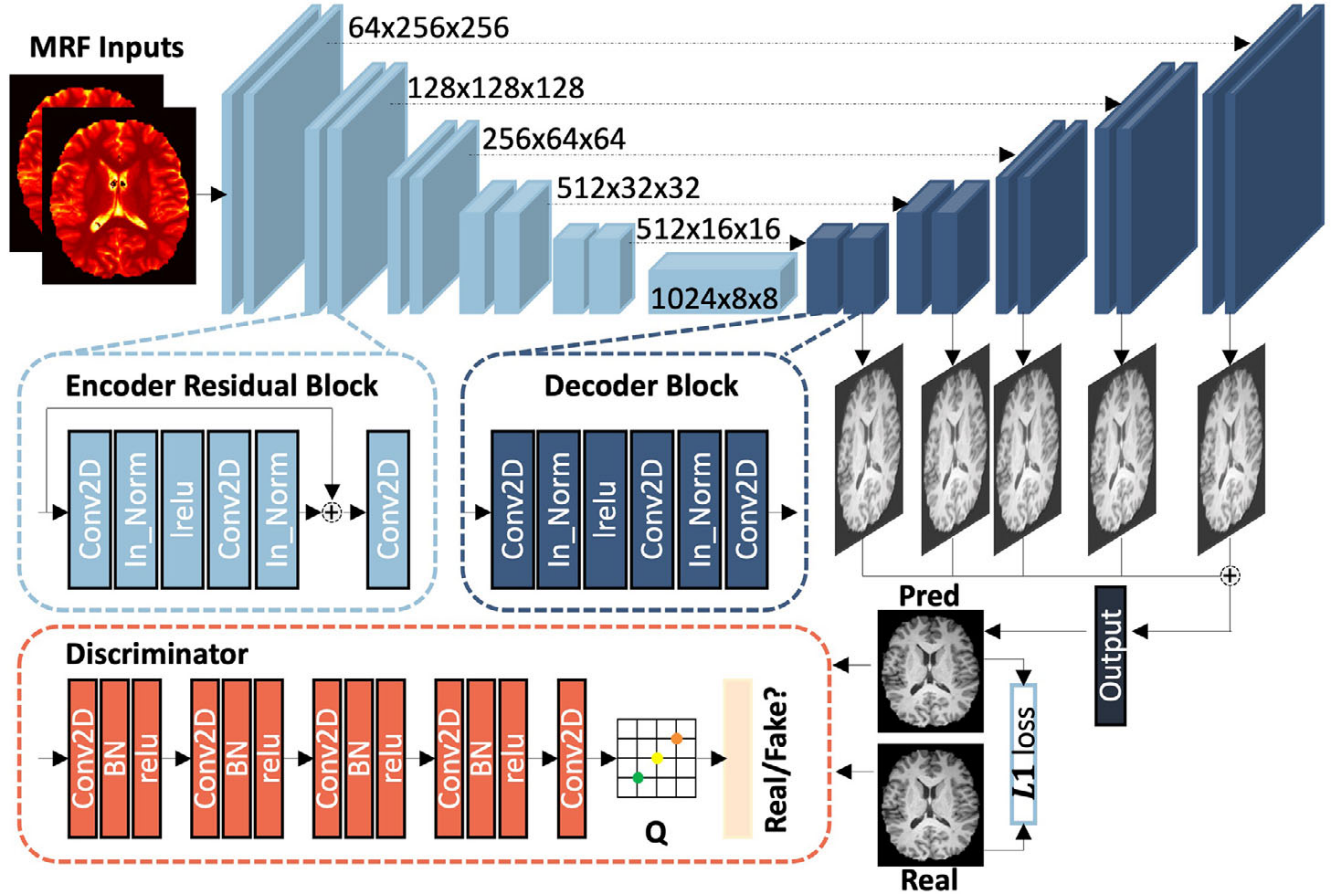
Architecture of the proposed QS‐Net. QS‐Net uses a GAN‐based design. The generator consists of a 6‐layer U‐Net backbone with residual connections in each encoder block and applies deep supervision in the decoding pathway to improve training stability. Numbers beside each block indicate the spatial dimensions at each encoding layer. The discriminator uses a PatchGAN architecture. Conv2D = two‐dimensional convolution; In_Norm = instance normalization; lrelu = leaky rectified linear unit; BN = batch normalization; relu = rectified linear unit.

The generator of QS‐Net (Figure 2) is based on a U‐Net backbone but incorporates key modifications: (1) Residual U‐Net Blocks: Unlike a standard U‐Net encoder, which uses simple convolutional blocks, our encoder pathway integrates residual connections within each block.^31^ This facilitates smoother gradient flow during backpropagation, mitigates vanishing gradients, and improves feature reuse, leading to more stable training and better convergence. (2) Deep Supervision: A critical addition is the application of deep supervision^32^ to the decoding pathway. Auxiliary output layers are attached to intermediate decoder stages, each computing a component of the primary L1 loss. This creates shorter paths for gradient propagation, encourages the learning of discriminative features at multiple scales early in training, and has been shown to reduce blurring in image synthesis tasks. (3) Instance Normalization & Leaky ReLU: We employ instance normalization^33^ and leaky ReLU activations^34^ within each block to stabilize training dynamics and enhance feature extraction. The discriminator adopts a standard PatchGAN architecture^35^ to focus on high frequency local realism.

#### 2.4.2 Two‐stage, pathology‐aware training strategy

To maximize generalizability, QS‐Net was trained in two distinct phases: (1) Pre‐training on Healthy Anatomy: The model was first trained end to end on data from healthy volunteers (Center A). This phase allows the network to learn a robust, foundational mapping from the quantitative, scanner‐independent T1/T2 MRF maps to the qualitative contrasts of conventional mpMRI. (2) Focused Fine‐tuning on Pathology: The pretrained model was then adapted to GBM patient data (Center B) using a frozen encoder strategy. Specifically, the encoder weights were made non‐trainable, and only the decoder weights were updated during fine‐tuning. This strategy forces the model to leverage the generalizable features learned from healthy anatomy in the first stage, while allowing the decoder to specialize in synthesizing the pathological features (e.g., tumor texture, edema) unique to the patient data.

#### 2.4.3 Implementation details

For both training phases, the two‐channel MRF T1 and T2 maps served as inputs to the network, while a single‐channel corresponding mpMRI images was used as target outputs. This identical input/output structure was maintained for all models in the comparative study. Five separate models were trained for each cohort, with each model dedicated to predicting a specific mpMRI sequence (T1w, T2w, T1‐FLAIR, T2‐FLAIR, or SWI). The L1 loss between the synthetic and ground‐truth mpMRI images was employed as the loss function for the synthesis network. For the PatchGAN‐based discriminator, mean squared error (MSE) loss was used to distinguish between real and synthetic patches. Model optimization was performed using the Adam algorithm with 200 epochs, with an initial learning rate set at 0.0002 that was reduced by a factor of 0.9 whenever the validation loss plateaued after the 100th epoch. The batch size was set to 1. All code was implemented in PyTorch and executed on an NVIDIA RTX 3090 graphics card.

### 2.5 Model evaluation

To ensure a fair and controlled comparison, all deep learning models evaluated in this study—including the baseline models (Res‐UNet, cGAN, Swin‐Transformer) and our proposed QS‐Net—were developed under an identical experimental framework. Specifically, each model was configured to accept the same two‐channel input (concatenated MRF‐derived T1 and T2 maps) and to generate a single‐channel output (one target mpMRI contrast: T1w, T2w, T1‐FLAIR, T2‐FLAIR, or SWI). Five separate instances of each model architecture were trained, one for each target contrast. All models were trained on the same dataset partitions, underwent the same preprocessing pipeline, and used the same primary voxel‐wise loss function (L1 loss) for the synthesis task. This setup isolates the impact of the network architecture on synthesis performance.

The U‐Net, conditional GAN (cGAN), and shifted‐window transformer (Swin Transformer) are state of the art architectures in MRI synthesis. U‐Net features a symmetric encoder‐decoder structure with skip connections, enabling precise localization and efficient feature extraction, and is widely used to generate high‐quality images by mapping between different MRI modalities.^10^ cGAN extends the traditional GAN framework by incorporating additional input information to guide both the generator and discriminator, allowing for the generation of realistic images conditioned on specific data, such as converting one MRI modality to another.^14^ The Swin‐Transformer is a hierarchical vision transformer that uses shifted windows for self‐attention, efficiently modeling both local and global image features while reducing computational complexity.^13^ To evaluate the effectiveness of our proposed QS‐Net, we conducted comprehensive comparisons with these state of the art models, assessing their performance across multiple MRI synthesis tasks using standardized metrics on both healthy volunteer and GBM patient datasets.

Three widely used evaluation metrics in medical image synthesis—mean absolute error (MAE), SSIM, and peak signal‐to‐noise ratio (PSNR)—were employed in this study to quantitatively assess model performance. The definitions of these metrics are provided below:

$$MAE=\frac{1}{N}\;\left|y\left(x\right)-g\left(x\right)\right|$$

$$MSE=\frac{1}{N}\;{\left(y\left(x\right)-g\left(x\right)\right)}^{2}$$

$$PSNR=10\log_{10}\left(\frac{L^{2}}{MSE}\right)$$

$$SSIM=\frac{\left(2\mu_{y\left(x\right)}\mu_{g\left(x\right)}+c1\right)\left(2\sigma_{y\left(x\right)g\left(x\right)}+c2\right)}{\left({\mu_{y\left(x\right)}}^{2}+{\mu_{g\left(x\right)}}^{2}+c1\right)\left(\sigma_{y\left(x\right)}^{2}+\sigma_{g\left(x\right)}^{2}+c2\right)}$$

Here, N represents the number of pixels in each image slice, while y(x) and g(x) refer to the ground truth and the synthetic mpMRI images, respectively. The terms $\mu_{y(x)}$ and $\mu_{g(x)}$​ denote the mean values, and $\sigma_{y(x)}$​ and $\sigma_{g(x)}$​ represent the variances of the ground truth and synthetic images. The covariance between y(x) and g(x) is indicated by $\sigma_{y(x)g(x)}$​. The constants c1​ and c2​ are included to prevent instability caused by a weak denominator, where $c1={\left(k_{1}L\right)}^{2}$ and $c2={\left(k_{2}L\right)}^{2}$. L specifies the dynamic range of pixel values. In this study, L was set to 4095, with $k_{1}$= 0.01 and $k_{2}$= 0.03 as default values.

A blinded qualitative evaluation was performed by two board‐certified radiologists (with 5 and 11 years of clinical experience, respectively) to assess the clinical acceptability of the synthetic images. The evaluation employed a double‐blind design: the radiologists were not informed of the image origin (synthetic or ground truth), and the images were presented in a randomized order. For each image (both from healthy volunteers and GBM patients), the radiologists independently scored three key perceptual metrics on a standardized 5‐point Likert scale (1 = worst, 5 = best): (1) Image Quality (IQ): the overall clarity, noise, and anatomical structure delineation, (2) Pathological/Anatomical Contrast (PC): the contrast and boundary definition for tumors in patients or gray/white matter in volunteers, and (3) Artifact Suppression (AS): the absence of motion, susceptibility, or registration artifacts.

### 2.6 Model generalizability comparison

To evaluate model generalizability, we designed a comparative experiment in which deep learning‐based image synthesis models were trained and tested using either conventional MRI or quantitative MRF as input data. Both types of models were developed using identical network architectures and training protocols to ensure a fair comparison. The models were trained with the healthy volunteer data acquired from center A and were assessed on external datasets not seen during training (GBM patient scans from center B). Quantitative metrics such as MAE, SSIM, and PSNR were used to evaluate IQ and anatomical fidelity. Additionally, qualitative assessments were performed to further measure the clinical relevance and consistency of the synthesized images.

### 2.7 Declaration of generative AI and AI‐assisted technologies in the writing process

During the preparation of this work the authors used ChatGPT‐4 in order to improve language and readability. After using this tool, the authors reviewed and edited the content as needed and take full responsibility for the content of the publication.

## 3 RESULTS

### 3.1 Quantitative evaluation

**FIGURE 3:**
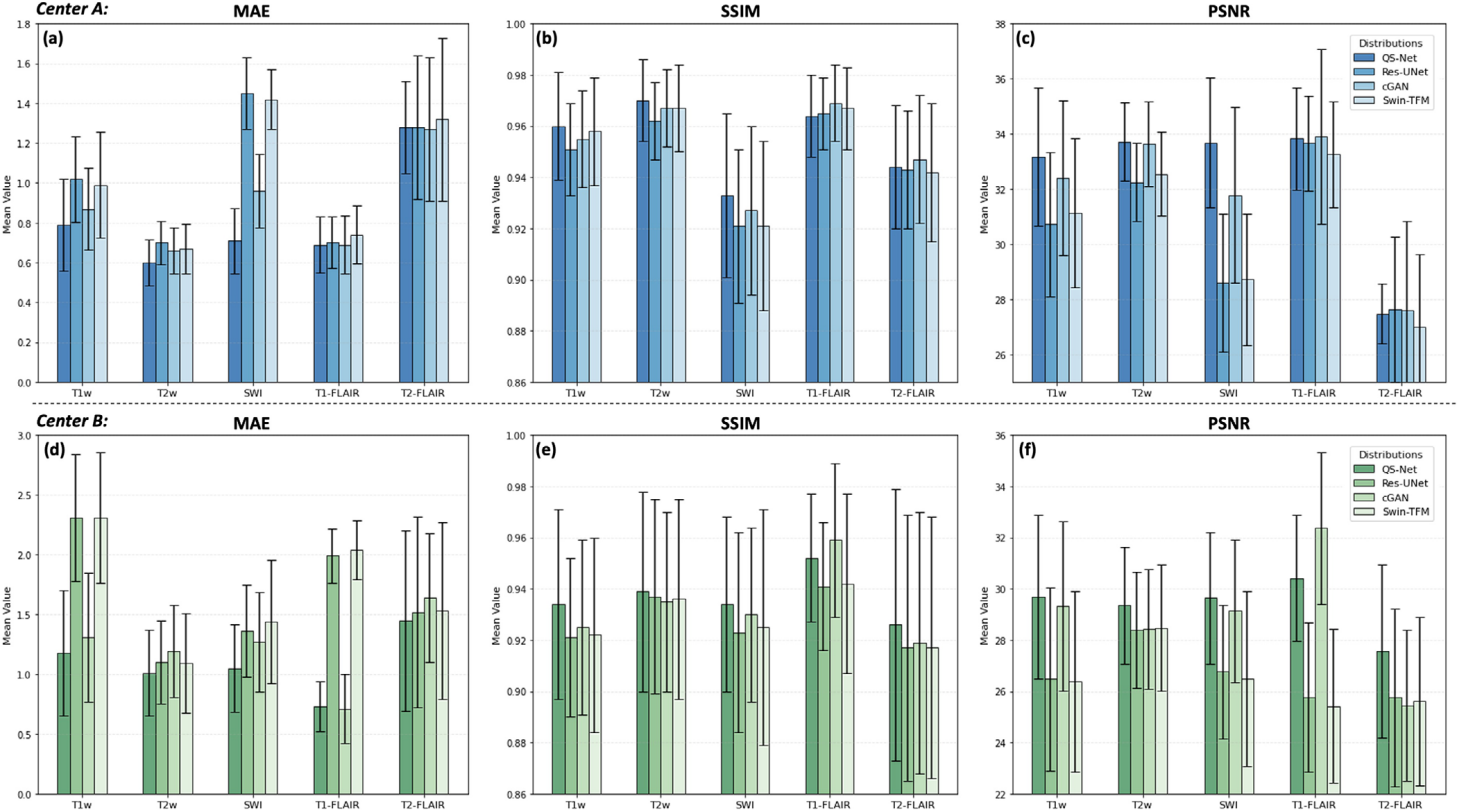
Quantitative error evaluation of deep learning models for mpMRI synthesis. Each subplot compares the performance of different models (indicated by the different colored bars) across five synthetic targets: T1w, T2w, SWI, T1‐FLAIR, and T2‐FLAIR (ordered from left to right within each subplot). The top row displays results from Center A: (a) Mean Absolute Error (MAE), (b) Structural Similarity Index (SSIM), and (c) Peak Signal‐to‐Noise Ratio (PSNR). The bottom row presents the corresponding metrics for Center B.

Figure 3 presents a quantitative comparison of the accuracy of synthetic mpMRIs generated by our proposed QS‐Net and other state of the art deep learning networks, using MAE, SSIM, and PSNR as evaluation metrics. Under the consistent experimental setup, QS‐Net demonstrated superior quantitative performance. For the 12 healthy volunteers from Center A, QS‐Net achieved significantly better performance than the other models in synthesizing T1w, T2w, and SWI images (*p* < 0.05). Although cGAN slightly outperformed QS‐Net in T1‐FLAIR and T2‐FLAIR synthesis, no statistically significant difference was observed (*p* > 0.05). For the 9 GBM patients from Center B, QS‐Net consistently outperformed the other models in T1w, T2w, SWI, and T2‐FLAIR synthesis across all metrics (*p* < 0.05), while cGAN performed marginally better in T1‐FLAIR synthesis.

Among the three baseline models, cGAN exhibited superior performance, achieving optimal MAE values in T2 FLAIR (healthy cohort) and T1 FLAIR (GBM cohort), alongside the highest SSIM scores for T1/T2 FLAIR sequences. In contrast, Swin‐TFM demonstrated intermediate performance, with metrics consistently falling between cGAN and Res‐UNet. Notably, Res‐UNet underperformed in healthy volunteer data, whereas no statistically significant difference was observed between Res‐UNet and Swin‐Transformer in pathological (GBM) data.

### 3.2 Qualitative evaluation

**FIGURE 4:**
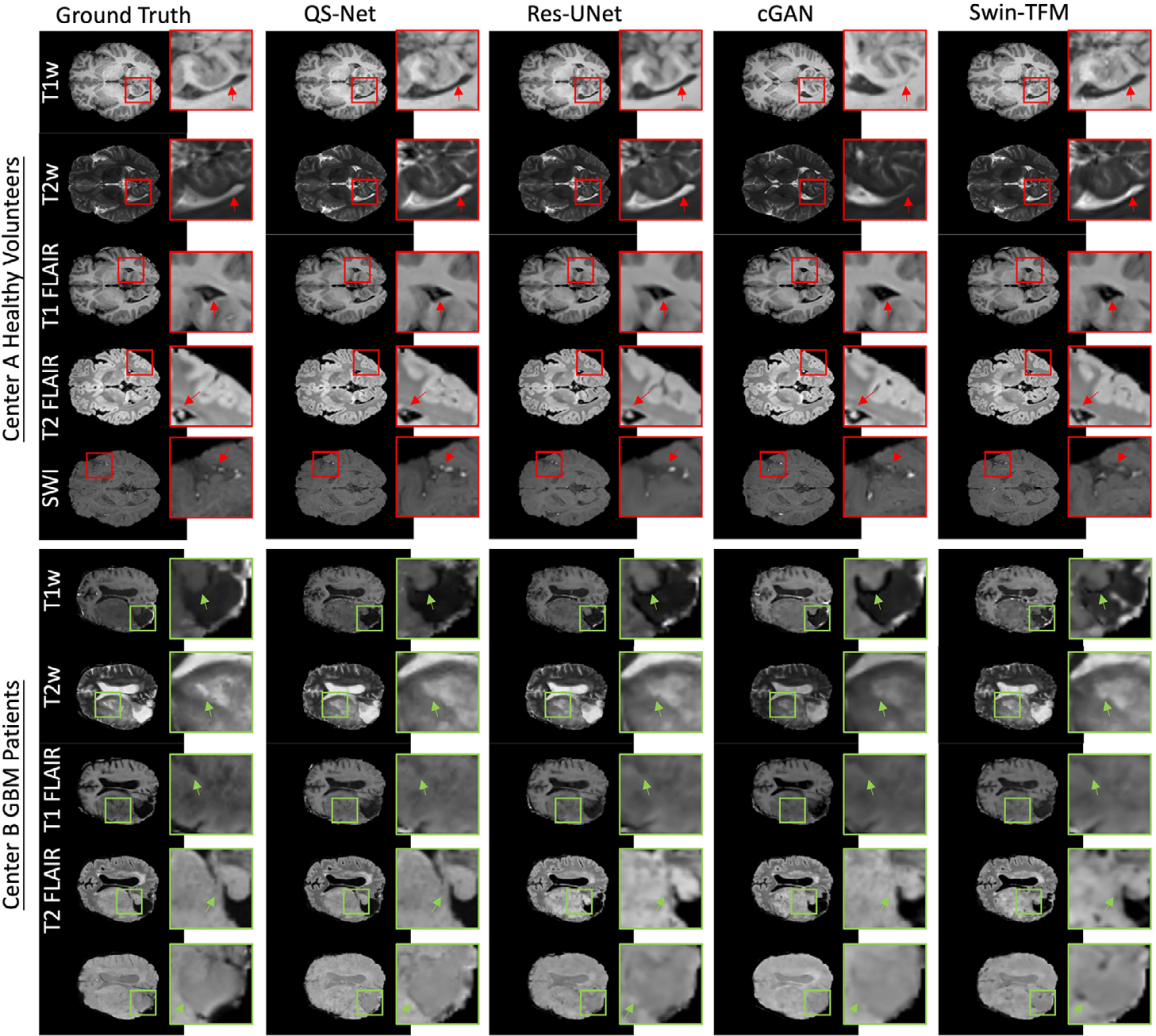
Visual comparison of ground truth and synthesized mpMRI images generated by different deep learning models. The top section shows results for a healthy volunteer from Center A; the bottom section shows results for a GBM patient from Center B. From left to right: ground truth mpMRI, and synthetic images from QS‐Net, Residual UNet, conditional GAN, and Swin Transformer. Each row within a section corresponds to a different mpMRI sequence: T1w, T2w, T1‐FLAIR, T2‐FLAIR, and SWI (top to bottom).

Figure 4 presents visual comparisons between the ground truth and the synthesized multiparametric MR images generated by different deep learning models for both a healthy volunteer and a GBM patient from the test cohort. For the T1w, T2w, and FLAIR images of the healthy volunteer, particular attention is given to the accuracy of anatomical structures such as the occipital horn of the lateral ventricle and the choroid plexus. In the SWI images of the healthy volunteer, the focus is on the cerebral arteries, which appear as hyperintensities. For the GBM patient, special attention is paid to the tumor regions.

The synthetic images from Res‐UNet were blurry with poor texture preservation, failing to accurately depict fine structures like the choroid plexus and cerebral arteries, and producing indistinct tumor boundaries. While the cGAN model achieved good overall visual quality, it misaligned tumor margins and omitted key anatomical landmarks. The Swin‐Transformer yielded results similar to Res‐UNet, with the poorest delineation of tumor texture and boundaries. In contrast, QS‐Net provided the closest visual approximation to the ground truth, excelling particularly in the depiction of tumor interface and textural details, which aligns with the superior quantitative performance.

The results of the blinded qualitative assessment by two radiologists are summarized in Table 2. For the healthy volunteer cohort (Center A), synthetic images achieved near‐indistinguishable quality from ground truth images across all metrics, with overall mean scores of 4.85 ± 0.34 for IQ, 4.73 ± 0.34 for AC, and 4.73 ± 0.30 for AS. These scores closely matched the ground truth values (IQ: 4.90 ± 0.21, AC: 5.00 ± 0.00, AS: 4.83 ± 0.29), demonstrating the model's high fidelity in synthesizing normal anatomical structures. For the GBM patient cohort (Center B), the synthetic images also performed well, receiving overall scores of 4.05 ± 0.48 (IQ), 4.35 ± 0.43 (PC), and 4.25 ± 0.61 (AS). While these scores were slightly lower than the corresponding ground truth (IQ: 4.40 ± 0.58, PC/AC: 4.50 ± 0.63, AS: 4.43 ± 0.65), they indicate that QS‐Net successfully preserved key diagnostic features, including tumor contrast and anatomical boundaries, with only a marginal reduction in perceived quality. These qualitative findings confirm the clinical acceptability of the synthetic images produced by QS‐Net and align with the superior quantitative performance reported earlier.

**TABLE 2:** Results of blinded qualitative evaluation by Radiologists.

	Center A (healthy volunteer)					
	Ground truth			Synthetic		
	R1	R2	Overall	R1	R2	Overall
IQ	4.85 ± 0.24	4.95 ± 0.16	4.90 ± 0.21	4.50 ± 0.33	4.85 ± 0.24	4.85 ± 0.34
PC/AC	5.00 ± 0.00	5.00 ± 0.00	5.00 ± 0.000	4.60 ± 0.40	4.85 ± 0.24	4.73 ± 0.34
AS	4.95 ± 0.16	4.70 ± 0.35	4.83 ± 0.29	4.65 ± 0.24	4.80 ± 0.35	4.73 ± 0.30
						

	Center B (GBM patient)					
	Ground truth			Synthetic		
	R1	R2	Overall	R1	R2	Overall
IQ	4.55 ± 0.60	4.25 ± 0.54	4.40 ± 0.58	4.15 ± 0.47	3.95 ± 0.50	4.05 ± 0.48
PC/AC	4.70 ± 0.63	4.30 ± 0.59	4.50 ± 0.63	4.55 ± 0.44	4.14 ± 0.34	4.35 ± 0.43
AS	4.55 ± 0.64	4.30 ± 0.67	4.43 ± 0.65	4.30 ± 0.63	4.20 ± 0.63	4.25 ± 0.61

Abbreviations: AS, Artifact Suppression; IQ, Image Quality; PC/AC, Pathological /Anatomical Contrast;R1, Rater 1; R2, Rater 2.

### 3.3 Cross‐vendor generalization: Quantitative versus qualitative inputs

**FIGURE 5:**
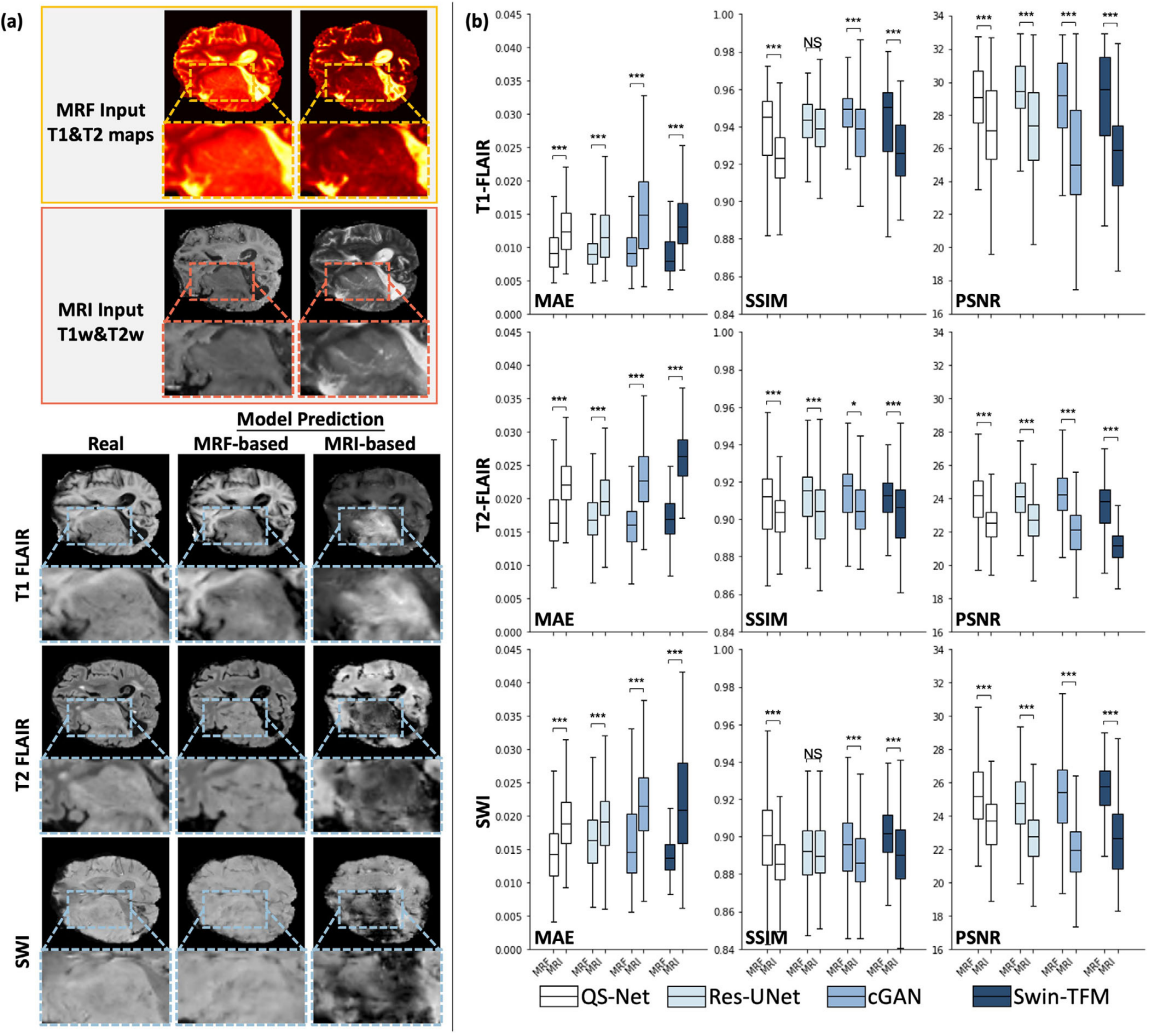
Generalizability evaluation of QS‐Net and other models. (a) Visual comparison of ground truth and synthesized mpMRIs generated by QS‐Net trained only on healthy volunteer data and tested on GBM patient data without finetuning. Two input types were used: MRF‐based and MRI‐based. (b) Quantitative comparison of prediction accuracy between MRF‐based and MRI‐based models across different architectures. Boxplot colors indicate model architectures; within each color, the left boxplot shows MRF‐based results and the right shows MRI‐based results. (*p*‐value: NS = non‐significant, * = *p* < 0.05, *** = *p* < 0.005).

To evaluate cross‐vendor generalization, Figure 5(a) shows visual comparisons between ground truth and synthetic images generated by models trained on Center A data and tested on Center B data without fine‐tuning. The models use the QS‐Net architecture with either standardized quantitative MRF maps or vendor‐specific qualitative MRI images as input. The MRF‐based model produces T1‐FLAIR and T2‐FLAIR images that closely match the ground truth, with tumor boundaries reasonably depicted. Although the predicted SWI image from the MRF‐based model differs visually from the ground truth, the tumor texture remains visible. In contrast, the MRI‐based model yields low‐quality images, with significant contrast differences compared to the ground truth. Tumor texture and boundaries are inadequately represented, and signal loss is evident in the synthesized T2‐FLAIR and SWI images of the MRI‐based model.

Quantitative comparisons of the prediction accuracy between MRF‐based and MRI‐based models using different model architectures are shown in Figure 5(b). For all three prediction targets (T1‐FLAIR, T2‐FLAIR, and SWI), no statistically significant differences were observed in the performances of MRF‐based models across different architectures. Among the MRF‐based models, the Swin‐Transformer model performs marginally better in synthesizing T1‐FLAIR images, while QS‐Net shows slightly better results for T2‐FLAIR and SWI synthesis. Importantly, the MRF‐based model consistently outperforms the MRI‐based model across all architectures and evaluation metrics (*p* < 0.005).

## 4 DISCUSSION

In the management of GBM, multiparametric MRI is essential for accurate tumor identification and assessment. While DL models have been developed to synthesize mpMRI contrasts to improve acquisition efficiency, these models typically rely on qualitative images as input, which introduces vendor‐ and protocol‐specific biases that limit their generalizability across different clinical sites. To address this, we developed a novel DL framework, the QS‐Net, designed to synthesize mpMRI from standardized quantitative T1 and T2 maps acquired via a single MRF sequence. Unlike conventional physics‐based synthetic MRI techniques, QS‐Net learns the mapping between quantitative parameters and qualitative contrasts in a data‐driven manner. Using multi‐institutional datasets, we demonstrate that this quantitative‐input approach enables superior cross‐vendor generalization compared to DL models trained on conventional qualitative images. In this discussion, we highlight the key findings of our study, examine potential underlying mechanisms, and propose future research directions for the community.

Quantitative evaluations demonstrated that our QS‐Net consistently outperformed the comparison networks in synthesizing T1w, T2w, and SWI images for healthy volunteers across all metrics (Figure 3(a‐c)), achieving the highest average MAE (0.79 ± 0.23, 0.60 ± 0.12, 0.71 ± 0.17), SSIM (0.960 ± 0.021, 0.970 ± 0.016, 0.933 ± 0.032), and PSNR (33.17 ± 2.49, 33.72 ± 1.41, 33.69 ± 2.36) for T1w, T2w, and SWI, respectively. For the GBM patient cohort, QS‐Net also achieved top‐ranked scores in most mpMRI modalities, with leading average MAE (1.18 ± 0.52, 1.01 ± 0.36, 1.05 ± 0.37, 1.45 ± 0.76), SSIM (0.934 ± 0.037, 0.939 ± 0.039, 0.934 ± 0.034, 0.926 ± 0.053), and PSNR (29.69 ± 3.21, 29.35 ± 2.29, 29.64 ± 2.58, 27.56 ± 3.39) for T1w, T2w, SWI, and T2‐FLAIR, respectively (Figure 3.(d‐f)). The superior performance of QS‐Net can be attributed to its specific architectural innovations. While prior studies have utilized U‐Nets or GANs for MRI synthesis,^10^, ^14^ QS‐Net integrates a deeply supervised residual U‐Net generator. The residual connections enhance training stability, while the deep supervision mechanism is crucial for preserving fine anatomical and textural details by enforcing multi‐scale feature consistency, directly addressing the over‐smoothing common in L1 loss‐based models. Furthermore, our two‐stage training strategy—pre‐training on healthy anatomy followed by decoder‐only fine‐tuning—is a novel methodological contribution for this task. It explicitly decouples the learning of a generalizable mapping from quantitative to qualitative imaging from the adaptation to disease‐specific features. We posit that this strategy is a key factor behind the model's exceptional cross‐center generalizability, as demonstrated in Figure 5. Although few studies have developed models to synthesize multiple MRI contrasts from a single input sequence—a task that mirrors clinical need—comparing our results with prior work focused on synthesizing a single target contrast highlights the effectiveness of our proposed QS‐Net. For example, Osman and Tamam reported a U‐Net‐based model trained on 305 brain tumor patients, achieving a highest mean SSIM of 0.946 for T2‐FLAIR synthesis.^10^ Dalmaz et al. proposed a Transformer‐based model trained on 35 and tested on 20 GBM patients, with T1w+T2w to T2‐FLAIR synthesis reaching an average SSIM of 0.886.^13^ The U‐Net‐based model, which relies mainly on L1 loss and a relatively simple architecture, tends to produce oversmoothed results and lacks the capacity to capture complex structural details inherent in MRI data. On the other hand, the Transformer‐based model, despite its advanced attention mechanisms, contains a large number of parameters, making it prone to underfitting when trained on relatively small datasets. The excessive model complexity of Transformer can hinder effective learning and generalization, resulting in suboptimal synthesis quality. In contrast, our QS‐Net achieves superior performance in mpMRI synthesis (Figure 3), largely due to its adaptation of deep supervision and adversarial loss. Deep supervision facilitates more effective gradient flow and multi‐scale feature learning, while adversarial loss encourages the generation of more realistic and high fidelity images by directly optimizing perceptual quality.

Interestingly, our model, trained exclusively on image data from healthy volunteers, demonstrated strong generalizability when tested on unseen GBM patient data, with predictions showing good agreement with the ground truth (Figure 5(a)). Notably, this superior cross‐vendor performance appears to be model‐independent, as networks using standardized quantitative MRF inputs consistently outperformed those using vendor‐specific qualitative MRI inputs across various architectures (Figure 5(b)). One possible explanation is that while absolute T1 and T2 values can vary across platforms, quantitative maps derived from a physics‐based technique like MRF provide a more consistent and standardized input. The MRF reconstruction process, which matches signals to a standardized dictionary, inherently reduces scanner‐specific biases relative to qualitative imaging. While MRF is not yet universally available, our results demonstrate the principle that models trained on such standardized quantitative inputs achieve superior cross‐vendor generalization compared to those trained on conventional qualitative images. Furthermore, the MRF dictionary, generated from Bloch equation simulations, encompasses a broad spectrum of signal evolutions, ensuring that even pathological changes in GBM are likely represented within the range encountered during training. As a result, the model can effectively generalize its learned mappings to new, unseen patient data. We selected MRF as the source of quantitative inputs not only for its single‐acquisition efficiency but, more critically, for its intrinsic standardization. While dedicated conventional sequences (e.g., inversion recovery spin echo for T1, multi‐echo spin echo for T2) can also generate quantitative maps, they often require longer, separate acquisitions and are more susceptible to site‐specific variations in hardware and calibration. The MRF framework, by matching signals to a physics‐based dictionary, provides a built‐in normalization that is less vendor‐ and protocol‐dependent. This property is fundamental for training models intended for cross‐center generalization, as evidenced by our results in Figure 5

Despite these promising findings, our study has several limitations. First, the QS‐Net model was trained and validated on a relatively small dataset, which may restrict the broader applicability of our results. The cohort size was constrained by the practical challenges of acquiring paired MRF and mpMRI in a clinical setting and was comparable to that of many pioneering deep learning‐based medical image synthesis studies (e.g., ∼15–50 patients).^12^, ^13^ While our hierarchical training and strict holdout testing mitigate overfitting, the results should be interpreted as a proof of concept. Future multi‐center studies with larger cohorts are needed to confirm generalizability, to establish clinical utility, and to address potential data bias.^36^ Secondly, our generalization comparison contrasted MRF‐derived quantitative maps with conventional qualitative images. While this demonstrates the advantage of a quantitative‐input framework, it does not isolate whether the benefit stems specifically from MRF's acquisition scheme or from the use of quantitative maps more generally. A future study directly comparing MRF‐based models to models trained on other conventional quantitative mapping sequences would be needed to disentangle these effects. Furthermore, we have not yet conducted a clinical evaluation of QS‐Net to verify the authenticity of the synthesized mpMRIs using a Turing test,^37^ nor have we assessed its effectiveness in tumor delineation. This lack of clinical validation means that the real‐world applicability and diagnostic reliability of QS‐Net remain unproven, highlighting the need for further studies and expert assessment. Additionally, our current synthesis model was developed using only axial slices, and its performance on coronal or sagittal views has not been evaluated, primarily due to the 2D acquisition scheme of the MRF sequence. To broaden the application of our network, incorporating 3D views of MRF and other mpMRI modalities will be necessary. These improvements are currently underway and will be considered as extensions of this study.

## 5 CONCLUSIONS

In this study, we developed a novel, generalizable mpMRI synthesis framework leveraging single‐sequence quantitative MRF for GBM patients. Our proposed QS‐Net demonstrated the ability to synthesize high fidelity mpMRIs and outperformed three state of the art networks. We validated that a synthesis model using standardized quantitative input maps demonstrates superior generalizability compared to conventional MRI‐based approaches. This work provides a framework for scanner‐agnostic mpMRI synthesis that can leverage emerging quantitative imaging techniques such as MRF. Further research involving larger patient cohorts and clinical evaluation is recommended to better contextualize and validate the findings of this study.

## CONFLICT OF INTEREST STATEMENT

The authors declare no conflicts of interest.
